# Evaluating the implementation of health and safety innovations under a regulatory context: A collective case study of Ontario’s safer needle regulation

**DOI:** 10.1186/1748-5908-8-9

**Published:** 2013-01-22

**Authors:** Andrea Chambers, Cameron A Mustard, Curtis Breslin, Linn Holness, Kathryn Nichol

**Affiliations:** 1 Dalla Lana School of Public Health, University of Toronto, 55 College Street, Toronto, ON, M5T 3M7, Canada; 2 Institute for Work and Health, 481 University Avenue, Suite 800, Toronto, ON, M5G 2E9, Canada; 3 Keenan Research Centre in the Li Ka Shing Knowledge Institute of St. Michael’s Hospital, 30 Bond Street, Toronto, ON, M5B 1W8, Canada; 4 Centre for Research Expertise in Occupational Disease, St. Michael's Hospital, 223 College Street, Toronto, ON, M5T 1R4, Canada; 5 University Health Network, 190 Elizabeth Street, Toronto, ON, M5G 2C4, Canada

**Keywords:** Safer engineered medical devices, Regulation, Implementation, Qualitative, Case study, Hospital

## Abstract

**Background:**

Implementation effectiveness models have identified important factors that can promote the successful implementation of an innovation; however, these models have been examined within contexts where innovations are adopted voluntarily and often ignore the socio-political and environmental context. In the field of occupational health and safety, there are circumstances where organizations must adopt innovations to comply with a regulatory standard. Examining how the external environment can facilitate or challenge an organization’s change process may add to our understanding of implementation effectiveness. The objective of this study is to describe implementation facilitators and barriers in the context of a regulation designed to promote the uptake of safer engineered medical devices in healthcare.

**Methods:**

The proposed study will focus on Ontario’s safer needle regulation (2007) which requires healthcare organizations to transition to the use of safer engineered medical devices for the prevention of needlestick injuries. A collective case study design will be used to learn from the experiences of three acute care hospitals in the province of Ontario, Canada. Interviews with management and front-line healthcare workers and analysis of supporting documents will be used to describe the implementation experience and examine issues associated with the integration of these devices. The data collection and analysis process will be influenced by a conceptual framework that draws from implementation science and the occupational health and safety literature.

**Discussion:**

The focus of this study in addition to the methodology creates a unique opportunity to contribute to the field of implementation science. First, the study will explore implementation experiences under circumstances where regulatory pressures are influencing the organization's change process. Second, the timing of this study provides an opportunity to focus on issues that arise during later stages of implementation, a phase during the implementation cycle that has been understudied. This study also provides the opportunity to examine the relevance and utility of current implementation science models in the field of occupational health where the adoption of an innovation is meant to enhance the health and safety of workers. Previous work has tended to focus almost exclusively on innovations that are designed to enhance an organization’s productivity or competitive advantage.

## Background

Safety-engineered medical devices (SEMD) are now widely recommended for the prevention of both needlestick and other sharp-related injuries in healthcare workplaces. Needlestick injuries arising from the unintentional puncture of the skin can result in the transmission of bloodborne pathogens between patients and healthcare workers. The World Health Organization has estimated that 2 million health-care workers annually are exposed to the risk of infectious disease transmission from a percutaneous exposure arising from a sharp device [1]. The process of being tested and receiving post-exposure treatment has been found to have psychological impacts on healthcare workers including generalized anxiety and post-traumatic stress disorder [2]. These injuries can also be costly with estimates for a single injury ranging from $65 to as high as $4,800 (2012 US$) for post-exposure treatment and testing [3]. As with many innovations, the adoption of SEMDs by healthcare organizations was slow initially [4] primarily due to the fact that the cost of SEMDs compared to conventional devices can be 25-80% higher [5].

This study will describe the implementation of SEMDs in healthcare institutions in the province of Ontario in response to a regulatory standard [6]. Regulation may serve as a potentially powerful institutional force to promote the adoption of occupational health and safety policies and practices [4,7]. There may also be limitations to the use of regulation when dealing with occupational health and safety issues [8]. Organizational compliance can be influenced by other motivators and conditions that impede or facilitate the adoption and implementation of the regulatory requirements [9].

Regulatory requirements to adopt the use of SEMDs have a primary objective to reduce the incidence of needlestick injuries among healthcare workers and the majority of studies that have examined safer needle regulation have focused on the prevention of needlestick injuries [10-12]. One consistent finding is that injuries associated with conventional sharps and SEMDs continue to occur. There are a few studies that have focused on examining issues associated with the integration of regulatory requirements [13-16]. Following the passage of legislation requiring the use of safety devices in British Columbia, Canada, an audit of sharps disposal containers was carried out in six hospitals [14]. This study found that conventional devices continued to be used and safety devices remained unactivated. To evaluate the implementation of the US Bloodborne Pathogen Standard, a compliance index was developed to examine organizational practices on a broad range of indicators [15]. Focusing on home health care workers, this study found that eighty percent of nurses reported limited access to SEMDs [15]. Very important information about the delivery of health and safety interventions can be drawn from studies that focus on describing regulatory compliance [17]. However, there is a need to understand the root causes of compliance issues and limitations in the design and delivery of system interventions.

This study will explore the implementation experience of acute care hospitals responding to safer needle regulation in Ontario through a collective case study design. The study will have the following objectives: 1) describe how acute care hospitals have responded and managed the integration of SEMDs under Ontario’s safer needle regulation, 2) to better understand the consequences of integrating SEMDs under Ontario’s safer needle regulation, and 3) to provide a contextualized understanding of remaining issues associated with the use and integration of SEMDs. Attention will be given to the challenges and processes associated with later stages of the implementation process including the integration of SEMDs into the norms and practices of the organization. Degree of implementation will be described by examining written policies and procedures, ongoing implementation activities that are in place, and whether these activities are perceived to support the use of SEMDs. The study is informed by concepts and theories from the implementation science literature.

### Theory: An Implementation Science Perspective

Klein and Sorra have defined implementation effectiveness as "the consistency and quality of targeted organizational members' use of [the] specific innovation" (p. 1058) [18]. When thinking about the adoption of safer needles, implementation effectiveness can be considered a state where conventional sharps have been replaced by safer alternatives. It may also include a state where there are clear expectations for the use of SEMDs, training is provided, workers value the added protection, and where workers consistently use the devices as intended. While implementation effectiveness models have identified organizational supports and processes that can promote the successful implementation of an innovation, less focus has been placed on describing additional external conditions that influence the implementation process. We have a limited understanding of the limitations and challenges experienced when adapting to regulatory requirements and the internal and external conditions that facilitate or impede change.

The organizational implementation effectiveness model modified for the healthcare sector [19] defines organizational factors thought to be most important in influencing implementation effectiveness. These factors include implementation climate, an implementation champion, clear and comprehensive implementation policies and procedures, innovation-values fit, management support, and financial resource availability [19]. While these factors have been found to be important in various settings and for different types of innovations [18-23], it has been recognized that implementation is not only influenced by internal organizational factors. We currently have very little information on how external conditions influence the implementation process including the requirements to meet regulatory standards [23]. Regulation may have a role in shaping the types of policies and procedures that are adopted or even how staff perceives the utility and value of an innovation [23]. Regulation may empower individuals within an organization who have already recognized the value of organizational change but have faced internal resistance from senior management. Regulatory changes may also result is a subsequent increase in available external resources to support implementation. In many ways, the presence of regulation can also present some additional challenges for organizations as they implement a new innovation. For example, regulatory requirements often define an effective date for the adoption and implementation of a new requirement that provides a narrow window for organizations to make necessary changes. This may prevent the use of a more complex implementation strategy.

**Figure 1:**
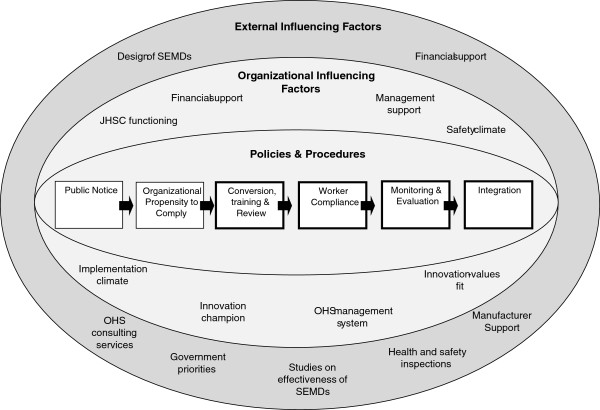
Proposed conceptual framework - Multi-level level influences on the implementation of safer engineered needles.

Figure 1 presents a conceptual framework for this study that highlights 1) the stages of the implementation process that will be examined and 2) the levels of influencing factors on the implementation process. The structure of this framework was influenced by a simplified conceptual model from the implementation science literature which emphasizes the importance of examining multi-level influences on the implementation process [24]. The stages of change defined at the core of the conceptual model were informed by consultations with program managers in the Ontario hospital sector in addition to guidelines for the implementation of SEMDs [25]. While the model defines six stages, this study focuses on the last four: conversion, training, and review; worker compliance; monitoring and evaluation; and integration. Due to the fact that the regulation has been in effect for 4 years, it was assumed that the majority if not all acute care hospitals in Ontario would have initiated some activities to implement SEMDs.

The stages of change defined at the core of the conceptual framework are surrounded by influencing factors on the implementation process. The selection of organizational and external influencing factors was influenced by the implementation effectiveness model [18,19] in addition to other concepts in the implementation science and occupational health and safety literature [4,26-35]. The conceptual model represents a starting point for thinking about the implementation process and conditions that may facilitate or impede the integration of SEMDs.

## Methods

### Design

A qualitative study is proposed. Understanding unique implementation experiences within different organizational contexts requires detailed description and an open-ended and discovery-oriented process [36]. The study is guided by a critical realist perspective [37]. This perspective carries specific assumptions that have implications for the design, field work, and analysis [38,39]. When following this perspective, there is a tendency to focus on structures, mechanisms and processes in context. The specific unit of analysis is seen as being influenced by multiple levels including institutional forces (e.g., regulation, organizational policies) and individual behavior (action, language, cognitive processes) [37]. Theory will play an important interactive role throughout the study framing the initial focus but also allowing for new emerging ideas and theoretical explanations.

The study will use a collective case study design [40]. The cases are instrumental to understanding collectively how Ontario’s safer needle regulation played out in acute care hospitals. The case study design is often used when the unit of analysis focuses on organizational change. In this study, a case study design allows the implementation experience to be examined within the broader context of the priorities, constraints and processes of the organization.

### Study sample

The study will focus on acute care hospitals in Ontario. Acute care hospitals were the first healthcare setting to be targeted by Ontario’s safer needle regulation; therefore, they have had a longer period of time to comply with the regulatory requirements. This provides an opportunity to focus on issues that arise during later stages of implementation, a period during the implementation cycle that has been understudied [24]. Second, focusing on acute care hospitals provides an opportunity to examine the implementation of SEMDs in a complex setting requiring the implementation of a broad range of different devices across different clinical departments where the types of healthcare workers, procedures and hazards will vary greatly.

The selection of organizations and the selection of informants will follow both a random and purposive sampling approach. The eligible sample of potential organizations includes all general or teaching hospitals that are within 40 kilometers from two offices held by the primary investigator. This includes 22 hospital sites, 3 of which are located in Ottawa and 19 in Toronto. The sample includes 8 teaching hospitals and 14 community hospitals. Hospital sites will be listed under three categories representing a) community hospitals in Toronto, b) teaching hospitals in Toronto and c) both teaching and community hospitals in Ottawa. Sites will be randomly selected from each category.

Organizations will be recruited with the assistance of consultants from a health and safety association in Ontario. At each hospital, the director of occupational health and safety will be contacted to discuss the study requirements and opportunities. Permission for the study to be carried out within the organization will be received from the organization's central gatekeeper (whose job title will vary between organizations). During this early recruitment phase, senior level employees and the organization's joint health and safety committee will be informed about the study.

At each site, information will be collected from two groups including organizational informants and front-line healthcare workers. Organizational informants are considered to be staff that had a direct role in the selection and integration of SEMDs which may include the director or manager of occupational health and safety, joint health and safety committee representatives, staff from professional practice, purchasing managers, infection control professionals and members of the sharps safety committee. The second group of participants will include healthcare workers with or without a direct safety role including unit managers and front line registered nurses. As hospitals have a very complex structure with multiple departments, interviews will be conducted with staff in two departments which will be selected in consultation with the director of occupational health and safety. Selection will be based on the extent to which safety needles are used on a regular basis within each department and other practical constraints (e.g., department restructuring, involvement in other ongoing research projects).

At each site, it is anticipated that 2-4 interviews will be conducted with organizational informants depending on the number of employees and groups directly involved in the implementation process. To identify workers within each department, a snowball sampling approach will be used where early informants at the senior and middle management level will send the recruitment invitation to other eligible employees. This study will consider ‘data saturation' (a stage where interviews are no longer providing new information or perspectives) as a primary criterion to decide when a sufficient number of interviews has been carried out [41,42]. It is anticipated that 8-10 interviews with front-line healthcare workers per site will be required to reach data saturation.

### Data collection

The implementation of SEMDs was a large and complex undertaking involving several organizational stakeholders at various levels within the organization. To help narrow the focus of the field work, an advisory committee was established with four individuals who had a key role in the development and implementation of Ontario’s safer needle regulation. The committee members were from regulatory, labour, and not-for-profit health and safety organizations. The role of the committee was to ensure the study was focused on the most pertinent issues and that key organizational informants were recruited. Through this consultation process a preliminary list of foreshadowed issues and topical questions about the case has been developed (Table 1). Foreshadowed issues serve as an initial conceptual structure defining what particular aspects of the case are of interest. They are meant to evolve overtime. Topical questions will also be used to guide the collection of more contextual information about the case.

**Table 1:** Foreshadowed issues and topical questions

**Foreshadowed Issues:**	
●	Does needlestick injury prevention continue to be a priority for organizations?
●	Are organizations continuing to improve the availability and quality of safety devices and monitoring issues with existing devices?
●	Are existing goals to eliminate needlestick injuries through the uptake of SEMDs realistic considering device availability and utility?
●	How have healthcare workers responded to the transition to SEMDs? Is there resistance to the use of these devices among specific groups of workers?
●	Has the regulation stimulated the uptake of safer engineered devices beyond the regulatory requirements?
●	Has the transition to SEMDs negatively impacted patient care in any way?
**Topical Questions:**	
Initial implementation procedures and activities:	
●	Was a sharps safety committee established?
●	Did the process involve inventory review?
●	What role did manufacturers have in facilitating the implementation process?
●	Were SEMD pilot tested?
●	Were workers involved in device selection?
●	What kind of training and education on SEMD use and needlestick injury prevention was provided?
●	Are there written policies and procedures in place?
Ongoing procedures and activities for sustained integration:	
●	Are there activities in place to increase compliance with the use of SEMDs?
●	Is the use of SEMDs monitored? How are issues addressed?
●	Is there continued training and education on the use of SEMDs and needlestick injury prevention?
●	Does the original sharps committee still meet?
●	Are SEMDs beyond the regulatory requirements being considered?
Contextual questions about the transition:	
●	When did the organization start to transition to the use of these devices?
●	Are there still exceptions to the use of SEMDs?
●	What do health and safety inspectors look for with regards to compliance with Ontario’s safer needle regulation
●	Has the joint health and safety committee had a role in the implementation process?
●	What are the organization’s ongoing health and safety priorities?

Data for this study will be collected via qualitative interviews and document analysis. Information will be collected from the three sites over a 9 month period. Field notes will be used to document the data collection process. The interviews will be guided by a semi-structured interview guide that will aim to ask broad questions about the implementation process and experience. The interview questions will evolve over the course of the study to accommodate the emerging findings of the case. Working with the informants in the department of occupational health and safety, any available supporting documents related to the organization’s strategy for implementing SEMDs will be reviewed. Relevant documents may include incident reports, newsletters, policies and procedures, meeting minutes, and program manuals.

### Analysis

The data for the analysis will include interview transcripts, field notes, and relevant organizational documents. Audio recordings will be transcribed verbatim by a transcriptionist and checked for accuracy by the research investigator.

The analysis will be treated as an ongoing process [43]. Case summary forms will serve as an analytical device and as a guide to the data collection process. Following each interview, a case summary form will be completed to highlight the main issues or themes identified from the initial review of the data and to summarize the information obtained. The foreshadowed issues and topical questions will influence the direction of the analysis including the content of the case descriptions [40]. The analysis can be described as following an interactive approach where both the empirical data and imported theory will constantly inform each other [43].

The analysis will have five key steps: immersion in the data, first level coding, second level coding, explanation and interpretation of categories, and cross-case analysis. During the first level coding process, descriptive codes will be applied to sections of data to summarize the data collected, assist with pattern identification and interpretation, and guide the identification of relevant text across interviews, documents and cases. First level coding will be followed by pattern coding [43,45]. Memoing will be used throughout the coding process as a strategy to identify and discuss promising codes that emerge from pattern coding and also to document the analysis process.

The general strategy will be to conduct analyses on each case individually before carrying out any cross-case analyses. The process of coding, memoing and documenting the analysis will be assisted with the use of NVivo (QSR International Pty Ltd. Version 9, 2010). The software will be used to help organize the large amount of data collected throughout the study, keep track of codes, and centralize and document the analysis process. The study aims to produce a descriptive case report for each site in addition to assertions based on the cross-case analysis.

Each case report will include an extensive description of each hospital’s strategy and contextualized experience of the integration of SEMDs highlighting specific issues and drawing on multiple perspectives from the interview informants. The analysis and interpretation process will generate some theoretical propositions regarding the relevance of existing concepts in the implementation science literature. A cross-case analysis will highlight themes and produce a series of assertions that will examine lessons learned about the implementation of SEMDs and the impact of the regulation.

## Discussion

It has been recognized that there is a need to better understand implementation from an ecological perspective examining the socio-political and organizational context [24]. Ontario’s safer needle regulation presents an interesting opportunity to study implementation of an occupational health and safety innovation in a complex environment and under a regulatory context. The decision to focus on Ontario’s safer needle regulation evolved from several stakeholder discussions with representatives who had a role in the initiation, design, implementation, and enforcement of the regulation. It was learned that the majority of target hospitals in Ontario have taken some action to respond to the requirements; however, it was also learned that implementation is as an ongoing process and several challenges remain.

Implementation has been described as having several stages. Fixsen has described these stages as: exploration and adoption, program installation, initial implementation, full operation, innovation and sustainability [24]. Sustained and successful implementation may include the continued commitment to the review and evaluation of safer alternatives. The design of SEMDs continues to improve. Initially, retrofitted devices were the most common type of safety devices. These devices are essentially a traditional syringe with a safety component added on such as a cap or sheath that slides or flips over the needle. Retrofitted devices are less optimal as effort is needed to activate the safety component. More advanced passive devices are now available including needles that will automatically retract into the barrel of the device. An organization might be categorized as being in a later implementation stage if they have continued to see the prevention of sharp injuries as an ongoing priority and continue to review more advanced safer needle technology.

Fixsen’s comprehensive review of the implementation science literature identified a paucity of literature that has been able to examine later stages of implementation [24]. Research tends to focus on the initial adoption of an innovation. Much less attention has been given to later stages of implementation including the integration of the innovation into the norms and practices of the organization. Focusing on the implementation of Ontario’s safer needle regulation presents a unique opportunity to examine issues and opportunities that emerge during the later stages of implementation including some of the barriers associated with the continued investment in more advanced safer needle technology.

There are many strengths in the design of this study. As described in the methods section, information will be collected from employees across the organization. When data collection strategies attempt to collect information from a larger number of organizations, input is often received from a single informant. It has been recommended that when looking at implementation one should obtain information from both management and front-line staff as these groups may have very different viewpoints and perceptions of organizational change [46].

The plan to obtain information from multiple informants and organizational documents was guided by an interest in examining the ‘degree of implementation’ [24]. Fixsen defines three levels of implementation including paper implementation, process implementation and performance implementation [24]. Paper implementation or the recorded theory of change includes the development of new policies and procedures. A study that focuses on paper implementation may only examine program documents to determine whether certain policies and practices are recorded. It is process implementation or the integrated theory of change that pertains to actually putting written policies and procedures into place. Performance implementation goes a step further to describe situations where procedures and processes have been put in place in such a way that they are perceived by employees to be useful and functional [24]. These concepts emphasize the importance of drawing on multiple informants and using both interviews and documents to obtain information on the organization’s commitment to the integration of SEMDs.

The substantive focus of this study also has important applications. There is a need to improve the implementation of public policies and programs in the field of health and safety. The move towards more system level interventions should be accompanied by pragmatic research to understand the consequences and limitation of these interventions. A report and recommendations from the Expert Advisory Panel’s review of Ontario’s health and safety system (2010) highlighted the importance of more pragmatic and participatory research projects with stakeholder input focusing on evaluation and emerging issues [47]. From these recommendations, a new prevention organization was formed under the Ministry of Labour in the province of Ontario. To move forward with a new occupational health and safety prevention mandate, implementation science and the barriers and facilitators to implementation should be of interest when decisions are being made regarding new legislation or enforcement priorities and the types of assistance that employers may require. We believe this study will make an important contribution by offering a more contextualized account of why occupational health and safety regulation may be challenging to implement for some organizations and what internal and external conditions facilitate or impeded the sustained integration of innovations.

It is also important to acknowledge the limitations of this work. While this study is focused on current issues and practices related to the integration of safer needle technology, there is also interest in the activities associated with the initial implementation of the regulatory requirements. It can be difficult to recall and describe events that are retrospective in nature [48]. This study will be collecting supplemental information from organizational documents including program manuals, written policies and procedures, training documents or meeting minutes to assist with the description of information regarding the timing and description of previous activities used to support the integration of SEMDs. It is also important to acknowledge that one’s interpretation of organizational changes may evolve overtime [49]. Describing the stage of implementation will be particularly important for contextualizing the informants’ reflections on the implementation experience.

With respect to the overall design, one of the most commonly referenced limitations associated with the case study approach is generalizability [50]. It is recognized that the findings of this study will not be generalizable in the probabilistic sense; however, it can be argued that the findings may be transferable. By bounding and situating the findings within a descriptive and contextual analysis, this allows the reader to decide whether the results can be transferable to their own situation. The nature of the descriptive and contextual elements of the case study approach is particularly useful in this respect. From another perspective, generalizability has been conceptualized from a uniquely qualitative processes that can be applied and further refined in new contexts [51,52].

There are several distinct theoretical and practical contributions of this study. A key research gap in the implementation science literature is a comprehensive understanding of the organizational and external context that impacts implementation [24]. In order to also enhance the potential practical applications of this work an advisory council with stakeholder representatives has been invited to influence both the direction of the study and the dissemination of the results. Information is being collected from employees across the organization to piece together a comprehensive understanding of the organization’s implementation experience. This information is being analyzed by bringing in an implementation science perspective, external implementation guidelines and experiences and strategies used by other sites. As this information could be very useful to the participating organizations for quality improvement purposes and for reflection, preparing individual case reports and giving presentations within each site will be an important practical contribution of this work.

## Abbreviations

SEMD: Safety-engineered medical device.

## Competing interests

The authors declare that they have no competing interests.

## Authors’ contributions

AC developed the idea for the study and led the development of the project proposal. The other authors, CM, CB, LH, KN, have served an advisory role throughout the process of developing the proposal and the stakeholder consultation phase. All authors read and approved the final manuscript.
